# Consequences of Delaying Surgical Intervention in Patients With Native Joint Septic Arthritis

**DOI:** 10.1093/ofid/ofaf662

**Published:** 2025-10-24

**Authors:** Takahiro Matsuo, Ryan B Khodadadi, Brian D Lahr, Omar M Abu Saleh, Pansachee Damronglerd, Jack W McHugh, Said El Zein, Brandon J Yuan, Gina A Suh, Aaron J Tande

**Affiliations:** Division of Public Health, Infectious Diseases, and Occupational Medicine, Department of Medicine, Mayo Clinic, Rochester, Minnesota, USA; Division of Public Health, Infectious Diseases, and Occupational Medicine, Department of Medicine, Mayo Clinic, Rochester, Minnesota, USA; Division of Infectious Diseases, Department of Medicine, Vanderbilt University Medical Center, Nashville, Tennessee, USA; Department of Biomedical Statistics and Informatics, Mayo Clinic, Rochester, Minnesota, USA; Division of Public Health, Infectious Diseases, and Occupational Medicine, Department of Medicine, Mayo Clinic, Rochester, Minnesota, USA; Division of Public Health, Infectious Diseases, and Occupational Medicine, Department of Medicine, Mayo Clinic, Rochester, Minnesota, USA; Division of Public Health, Infectious Diseases, and Occupational Medicine, Department of Medicine, Mayo Clinic, Rochester, Minnesota, USA; Division of Public Health, Infectious Diseases, and Occupational Medicine, Department of Medicine, Mayo Clinic, Rochester, Minnesota, USA; Department of Orthopedic Surgery, Mayo Clinic, Rochester, Minnesota, USA; Division of Public Health, Infectious Diseases, and Occupational Medicine, Department of Medicine, Mayo Clinic, Rochester, Minnesota, USA; Division of Public Health, Infectious Diseases, and Occupational Medicine, Department of Medicine, Mayo Clinic, Rochester, Minnesota, USA

**Keywords:** desirability of outcome ranking (DOOR), native joint septic arthritis (NJSA), treatment failure

## Abstract

**Background:**

Although recent guidance recommends early surgical debridement for native joint septic arthritis (NJSA), supporting data, particularly on long-term outcomes, remains scarce.

**Methods:**

We conducted a retrospective multicenter cohort study of adults (≥18 years) with NJSA who underwent surgery across Mayo Clinic campuses between 2012 and 2021. Clinical outcomes at 1 year were assessed using a 9-level Desirability of Outcome Ranking (DOOR) scale, incorporating survival, treatment failure (relapse, reinfection, readmission, or significant surgical events), and joint recovery. Time to surgery from hospital admission was analyzed both as a continuous variable and as categories: < 1, 1–2, or ≥3 days.

**Results:**

Among 268 patients, 30% underwent surgery <1 day from admission, 47% in 1–2 days, and 24% in ≥3 days. At 1 year, 57% achieved full recovery without unfavorable events (DOOR score 1), while treatment failure occurred in 34%. In unadjusted analyses, longer surgical delay was significantly associated with higher (worse) DOOR scores (per IQR increase [from 0 to 2 days], OR: 1.5; 95% CI: 1.0–2.1; *P* = .026), increased 1-year mortality (HR: 1.7; 95% CI: 1.1–2.6; *P* = .019) and treatment failure (HR: 1.5; 95% CI: 1.1–2.0; *P* = .007). Even after adjusting for age and Charlson comorbidity index, the association between surgical delay and treatment failure remained significant (HR: 1.5; 95% CI, 1.1–2.0; *P* = .016).

**Conclusions:**

The finding that delayed surgical intervention is associated with an increased risk of treatment failure reinforces current expert recommendations for timely surgical management in NJSA.

## Teaser

Delayed surgery in native joint septic arthritis was associated with higher treatment failure at 1 year. The DOOR-based analysis supports early intervention to improve long-term outcomes.

## Graphical Abstract

**Graphical Abstract:**
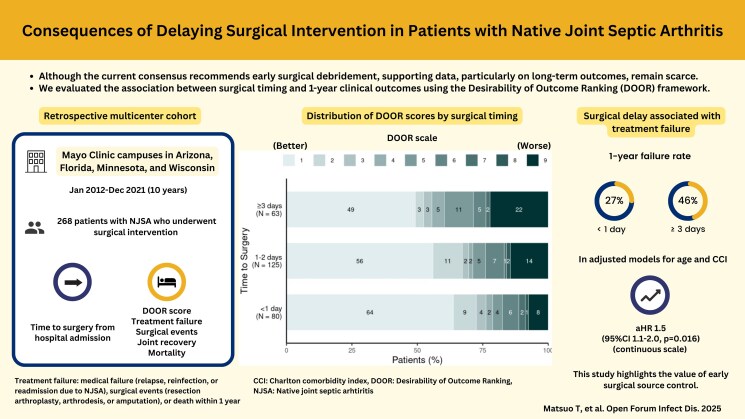

Native joint septic arthritis (NJSA) is a severe musculoskeletal infection that can result in joint damage, long-term functional impairment, systemic complications, and even death [1–5]. Optimal outcomes depend on early recognition and effective management, which typically includes targeted antimicrobial treatment with surgical or procedural source control including serial needle aspiration with lavage, arthroscopic drainage, and open arthrotomy [5–9].

Recent expert guidance recommends surgical lavage or repeat joint aspiration, depending on the hospital's available resources [8, 9]. Specifically, the 2023 Surgical and Antibiotic Treatment of Native Joint Septic Arthritis (SANJO) consensus [9], recommends surgical intervention within 24 h of diagnosis to avoid the need for repeat surgical debridement, noting that delays beyond 24 to 48 h may increase this risk [9]. However, this recommendation is classified as low quality with a weak evidence base (C2), and robust data on the long-term impact of delayed surgical intervention is sparse.

Previous studies have reported overall treatment outcomes of NJSA, including mortality, recurrence of infection, readmission, and major surgical events such as joint resection, arthrodesis, and amputation [1, 5, 7, 10]. Although several studies have also focused on a potential association between delayed surgery and adverse clinical outcomes—including lower treatment success rates [11], poorer functional recovery, and increased need for repeat surgical interventions [12]—a large-scale cohort study is needed to further elucidate the impact of surgical timing on long-term clinical outcomes.

The Desirability of Outcome Ranking (DOOR) framework has been proposed to evaluate interventions by integrating multiple clinically important outcomes into a single, patient-centered measure [13]. Instead of focusing on a single endpoint, DOOR ranks the overall desirability of clinical outcomes by combining multiple dimensions of treatment benefits and unfavorable outcomes [14]. This approach provides a more comprehensive and clinically meaningful assessment of treatment impact and has been increasingly applied in the major infectious diseases studies, including those on *Staphylococcus aureus* bacteremia [15], urinary tract infections [16], intraabdominal infections [17], multidrug-resistant pathogens [18], and candidemia [19].

In this study, we applied the DOOR methodology to our cohort of patients with NJSA. Our objective was to evaluate the association between time to surgical intervention and clinical outcomes, particularly long-term outcomes at 1 year, with the goal of proposing future practice and guideline development regarding the timing of operative management in NJSA.

## METHODS

We conducted a multi-center retrospective cohort study of adult patients (age ≥18 years) diagnosed with NJSA who underwent surgical intervention across Mayo Clinic campuses in Arizona, Florida, Minnesota, and Wisconsin from January 2012 to December 2021. This study builds upon a previously published cohort of NJSA cases (321 joints from 299 patients) [6].

### Data Collection and Definitions

Patients were initially identified using International Classification of Diseases (ICD) 9 and 10 diagnosis codes associated with NJSA (711, 098, 036, 003, M00–M01, A01, A18, A39, A54, A69, and B06) from an institutional data search tool. All identified cases were further reviewed manually to confirm the diagnosis using modified Newman criteria [20], which require one or more of the following: (1) isolation of a pathogenic organism from the affected joint; (2) isolation of a pathogen from another sterile site (eg, blood) with a clinically suspected septic joint; (3) turbid joint fluid with typical clinical features in the setting of prior antibiotics; or (4) pathologic or postmortem features consistent with septic arthritis. Patients were excluded if they had prosthetic joint infections, retained hardware or prior surgical excision of the affected joint, inadequate medical records, alternative diagnosis, or declined research authorization under Minnesota statute.

Clinical data were manually abstracted from electronic medical records and managed using REDCap electronic data capture tools hosted at Mayo Clinic [21]. Variables collected included demographics, comorbidities, clinical features, diagnostics, microbiologic findings, surgical approach, antimicrobial therapy, and clinical outcomes. The CCI was calculated with age-adjustment [22]. Time to surgery was defined as the number of days between the date of hospital admission and the first surgical intervention for NJSA.

### Outcome Measures

The primary outcome was clinical status at 1 year, assessed using a modified DOOR ordinal scale (Table 1). This scale ranged from level 1 (alive, event-free, and fully recovered) to level 9 (death), incorporating a multidimensional assessment of patient outcomes including survival status, treatment failure, surgical events, and functional recovery into a single ordinal measure. The DOOR framework was developed by adapting a previously established model for prosthetic joint infection (PJI) [23]. The initial version was drafted by TM and AT and subsequently refined through discussion with two additional musculoskeletal infection experts (OA and GS) and one orthopedic surgeon (BY) to better reflect the specific clinical context of NJSA. The modified DOOR scale incorporates treatment failure into the outcome ranking, defined as a composite of any of the following events: medical failure—including relapse (defined as NJSA of the previously infected joint with the same organism or culture-negative), reinfection (NJSA of the same joint caused by a different organism), or readmission due to NJSA; surgical events—including resection, arthroplasty, arthrodesis, or amputation; or death from any cause. This is consistent with the definitions of treatment failure from several prior studies [3, 5]. Among 1-year survivors, recovery was categorized as “full” (indicating return to baseline or near-baseline function) or “qualified” (defined by clinically significant residual disability, such as persistent pain, reduced range of motion, or need for mobility aids) [23, 24]. Secondary outcomes included individual components of the DOOR scale over 1-year follow-up: mortality, treatment failure, and surgical events.

**Table 1.** Desirability of Outcome Ranking (DOOR) Ordinal Scale

Rank	Alive	Failure^a^	Surgery	Recovery^b^	Reason For Failure
1	Yes	No	Native	Recovery	NA
2	Yes	No	Native	Qualified recovery	NA
3	Yes	Yes	Native	Recovery	Medical failure only
4	Yes	Yes	Native	Qualified recovery	Medical failure only
5	Yes	Yes	Resection or Arthroplasty	Recovery	Surgical event (± medical failure)
6	Yes	Yes	Resection or Arthroplasty	Qualified recovery	Surgical event (± medical failure)
7	Yes	Yes	Arthrodesis	NA	Surgical event (± medical failure)
8	Yes	Yes	Amputation	NA	Surgical event (± medical failure)
9	No	Yes	NA	NA	Death

Abbreviation: NA, not applicable.

^a^Failure was defined as a composite of relapse, reinfection, readmission due to NJSA, surgical events, or death.

^b^Qualified recovery was defined by clinically significant residual disability, such as persistent pain, reduced range of motion, or need for mobility aids.

## STATISTICAL ANALYSIS

Baseline characteristics were described by percentage and number of patients for categorical variables and by median and interquartile range (IQR) for continuous variables. Clinical outcomes were described similarly, except mortality and treatment failure were both treated as time-to-event outcomes and summarized using the Kaplan-Meier estimator. To analyze unadjusted associations between surgical delay and each of the outcomes, separate univariable regression models were fitted so that the effect of delay could be assessed in continuous (and potentially non-linear) fashion and after categorization to three intervals (<1, 1–2, ≥ 3 days). Continuous values of delay time were initially modeled using a restricted cubic spline function with 4 default knots to assess linearity assumptions. Because of extreme rightward skewed values, we first transformed this variable by calculating the log (delay + 1) after truncating values at 15 days (ie, all values greater than 15 were re-coded to 15) before applying the spline function to the data. We used the proportional odds ordinal logistic model to analyze the 1-year DOOR ordinal outcome scale and the Cox proportional hazards model for time to event (ie, death, treatment failure) outcomes during 1-year follow-up; additionally, as a subset analysis of 1-year survivors, we applied the binary logistic model for analyzing qualified/full recovery at 1 year. These regression analyses were then repeated after adjusting for age and CCI. Overall, the models showed little to no evidence of non-linear effects of delay on the log scale according to likelihood ratio tests, suggesting an approximately linear relationship with the log odds/hazard for the outcomes. The proportional odds assumption of the ordinal logistic model was evaluated by fitting separate binary logistic models for each possible dichotomization of the DOOR outcome and then examining the uniformity of the estimated odds ratios across the full range of cutoffs. Three plots were generated to assess the validity of this underlying assumption, one for each functional form considered for time to surgery: (1) continuous with non-linear effect, (2) continuous with linear effect, and (3) unordered categorical (3-level) effect. As illustrated in Supplementary Figure 1, the model assuming a linear functional form provided the most satisfying results, whereas the assessment of the non-linear model fit suggested the proportional odds assumption was questionable due to an apparent increasing effect of time with increasing ordinal levels of DOOR. Therefore, we focused on assessing delay, as it relates to clinical outcomes, on a continuous scale assuming a linear relationship (after logarithmic transformation). However, the outcome comparisons between delay time intervals were also done and reported for the sake of completeness. All analyses were conducted in R version 4.4.1 (R Core Team, 2024) and used 0.05-level significance tests.

## RESULTS

### Baseline Demographic and Clinical Characteristics

A total of 268 adult patients with NJSA who underwent surgical intervention met the study criteria. The median age at time of surgery was 62.3 years (interquartile range [IQR], 53.7–73.8), and 61% (*n* = 164) were male. The median CCI was 2 (IQR, 0–4). Diabetes mellitus was present in 33% (*n* = 89) of patients, and chronic kidney disease was noted in 19% (*n* = 51). A total of 30 patients (11%) were immunocompromised, and 15% (*n* = 40) had a history of non-dermatologic malignancy. Pre-existing arthropathy of the affected joint was present in 38% of patients (*n* = 101), with osteoarthritis accounting for 86% (*n* = 86/101) of these cases. The median duration of symptoms prior to presentation was 4 days (IQR, 2–13). The most commonly affected joint in these patients was the knee (43%), followed by the shoulder (14%), hip (12%), wrist (12%), and ankle (9%). Polyarticular involvement was infrequent, occurring in 11% of patients (*n* = 29) (Table 2).

**Table 2.** Baseline Demographic and Clinical Characteristics

Characteristic	*N*	Overall (*N* = 268)
Age at time of surgery, y	268	62.3 (53.7, 73.8)
Male sex	268	164 (61%)
Race	262	…
American Indian/Alaska Native	…	2 (1%)
Asian	…	6 (2%)
Black or African American	…	6 (2%)
White	…	248 (95%)
Body mass index, kg/m²	268	29.8 (25.2, 34.7)
Charlson comorbidity index (severity-weighted)	268	2 (0, 4)
Charlson comorbidity index (age- and severity-weighted)	268	4 (2, 7)
Smoking status	268	…
Never	…	151 (56%)
Former	…	82 (31%)
Current	…	35 (13%)
Alcohol dependence	268	40 (15%)
Intravenous drug use	268	7 (3%)
Pre-existing arthropathy of affected joint	267	101 (38%)
Osteoarthritis of affected joint	…	86 (86%)
Crystalline arthropathy of affected joint	…	8 (8%)
Prior inflammatory rheumatic disorder involvement of affected joint	…	9 (9%)
Previous native joint septic arthritis of affected joint	…	4 (4%)
Diabetes	268	89 (33%)
Inflammatory arthropathy	268	…
No	…	243 (91%)
Rheumatoid arthritis	…	19 (7%)
Other	…	6 (2%)
Chronic kidney disease	268	51 (19%)
End stage renal disease/dialysis	268	16 (6%)
History of non-dermatologic malignancy	268	39 (15%)
Other immunocompromised condition^a^	268	30 (11%)
Predisposing dermatologic condition	268	17 (6%)
Duration of symptoms at presentation	266	4 (2, 13)
Number of joints involved	268	…
1	…	239 (89%)
2	…	22 (8%)
≥ 3	…	7 (3%)
Joint Type	268	…
Knee	…	115 (43%)
Glenohumeral	…	37 (14%)
Hip	…	31 (12%)
Wrist	…	31 (12%)
Ankle	…	25 (9%)
Elbow	…	12 (4%)
Metacarpophalangeal	…	5 (2%)
Interphalangeal	…	3 (1%)
Acromioclavicular	…	2 (1%)
Metatarsophalangeal	…	2 (1%)
Likely source of infection	187	…
Hematogenous	…	128 (68%)
Traumatic	…	24 (13%)
Iatrogenic	…	14 (7%)
Other	…	21 (11%)
Blood cultures positive	239	109 (46%)
Culture result	201	…
Methicillin-susceptible *Staphylococcus aureus*	…	104 (52%)
Methicillin-resistant *Staphylococcus aureus*	…	15 (7%)
*Staphylococcus lugdunensis*	…	2 (1%)
Coagulase negative Staphylococci	…	6 (3%)
*Streptococcus pyogenes*	…	6 (3%)
*Streptococcus agalactiae*	…	14 (7%)
Other beta-hemolytic Streptococci	…	22 (11%)
*Streptococcus pneumoniae*	…	4 (2%)
Other Streptococcus species	…	1 (0%)
*Enterococcus faecalis*	…	2 (1%)
*Neisseria gonorrhoeae*	…	1 (0%)
Pasteurella species	…	2 (1%)
*Escherichia coli*	…	3 (1%)
Other Enterobacterales	…	5 (2%)
*Pseudomonas aeruginosa*	…	2 (1%)
Other	…	12 (6%)
Polymicrobial	268	14 (5%)
Time from admission to surgery	268	…
< 1 d	…	80 (30%)
1–2 d	…	125 (47%)
≥ 3 d	…	63 (24%)
Median (IQR), days	…	1 (0, 2)
Surgical approach	268	…
Arthroscopic	…	130 (49%)
Open arthrotomy	…	128 (48%)
Needle lavage	…	5 (2%)
Other	…	5 (2%)
Duration of antimicrobials, days	267	39 (28, 46)

Abbreviation: IQR, interquartile range.

Values represent median, lower quartile, and upper quartile for continuous variables, whereas number and percentage of patients are presented for categorical variables. *N* is the number of non-missing values.

^a^Defined as any of the following: corticosteroid use equivalent to more than 20 mg of prednisone daily for over 2 weeks; use of other immunosuppressive agents; human immunodeficiency virus/acquired immunodeficiency syndrome (HIV/AIDS); primary immunodeficiency; or a history of solid organ or hematopoietic stem cell transplantation.

Blood cultures were positive in 46% (109/239) of patients with cultures obtained. Among 201 cases with available joint culture data, *S. aureus* was the most frequently isolated organism (52% methicillin-sensitive *S. aureus*, 7% methicillin-resistant *S. aureus*), followed by β-hemolytic streptococci (21%, *n* = 42). Gram-negative organisms were less commonly identified (6%, *n* = 12).

Surgical intervention was performed <1 day, 1–2 days, and ≥3 days from hospital admission in 30% (*n* = 80), 47% (*n* = 125), and 24% (*n* = 63) of patients, respectively; the median time to surgery was 1 day (IQR, 0–2). Among the 63 patients with delayed intervention (≥3 days), reasons for delay included diagnostic uncertainty (32%), hemodynamic instability (19%), prioritized management of concurrent infections such as endocarditis or discitis (13%), orthopedic surgeons’ decision/scheduling (10%), failed initial medical management (5%), anticoagulation (3%) (Supplementary Table 1). Arthroscopic debridement (49%) and open arthrotomy (48%) were performed in nearly equal proportions, whereas needle lavage was used infrequently (2%). The median duration of antimicrobial therapy was 39 days (IQR, 28–46).

### Clinical Outcomes at 1 Year

At 1 year after the surgical intervention, 57% of patients (*n* = 152) achieved the best possible outcome of the DOOR ordinal scale (level 1). In contrast, 14% (*n* = 38) died within 1 year highlighting the worst possible outcome (level 9). The median DOOR score was 1 (IQR, 1–5). Among the 230 one-year survivors, 78% (*n* = 179) achieved full recovery, whereas 22% (*n* = 51) experienced qualified recovery, including ongoing pain (*n* = 39), limited range of motion (*n* = 23), and/or need for mobile aids (*n* = 13), with some patients meeting more than one criterion. At 1 year, 34% (*n* = 92) met the criteria for treatment failure, including death (*n* = 38), relapse (*n* = 30), reinfection (*n* = 2), readmission (*n* = 27), or significant surgical events (*n* = 40). The cumulative 1-year rates of resection or arthroplasty, arthrodesis, and amputation were 13% (*n* = 33), 2% (*n* = 4), and 1% (*n* = 3), respectively (Table 3).

**Table 3.** Clinical Outcomes

Outcome	Overall (*N* = 268)
DOOR outcome scale at 1 y	…
1	152 (57%)
2	23 (9%)
3	8 (3%)
4	7 (3%)
5	16 (6%)
6	17 (6%)
7	4 (1%)
8	3 (1%)
9	38 (14%)
Median (IQR)	1 (1–5)
Treatment side effects	…
None	249 (93%)
Minor	16 (6%)
Major	3 (1%)
Recovery at 1 y *(N* = *230 one-y survivors)*	…
Full	179 (78%)
Qualified^a^	51 (22%)
Ongoing pain, *n*	39
Limited range of motion, *n*	23
Need for mobile aids, *n*	13
Mortality	…
90-d cumulative event rate	19 (7%)
1-y cumulative event rate	38 (14%)
Treatment failure	…
90-d cumulative event rate	55 (21%)
1-y cumulative event rate^a^	92 (34%)
Death, *n*	38
Relapse, *n*	30
Reinfection, *n*	2
Readmission, *n*	27
Surgical events, *n*	40
Resection or arthroplasty	…
90-d cumulative event rate	16 (6%)
1-y cumulative event rate	33 (13%)
Arthrodesis	…
1-y cumulative event rate	4 (2%)
Amputation	…
1-y cumulative event rate	3 (1%)

Abbreviation: IQR, interquartile range.

The nine-category ordinal DOOR outcome scale, along with the treatment side effect and recovery outcomes, are reported as simple percentages, while time-to-event outcomes are assessed as cumulative event rates calculated as a function of time after the index surgery using the Kaplan-Meier estimator.

^a^Some patients with events for the combined end point had more than 1 type of event during the 1-year follow-up, resulting in column numbers that sum to greater than the reported total number with composite events.

### Association Between Surgical Timing and Outcomes

**Figure 1.**
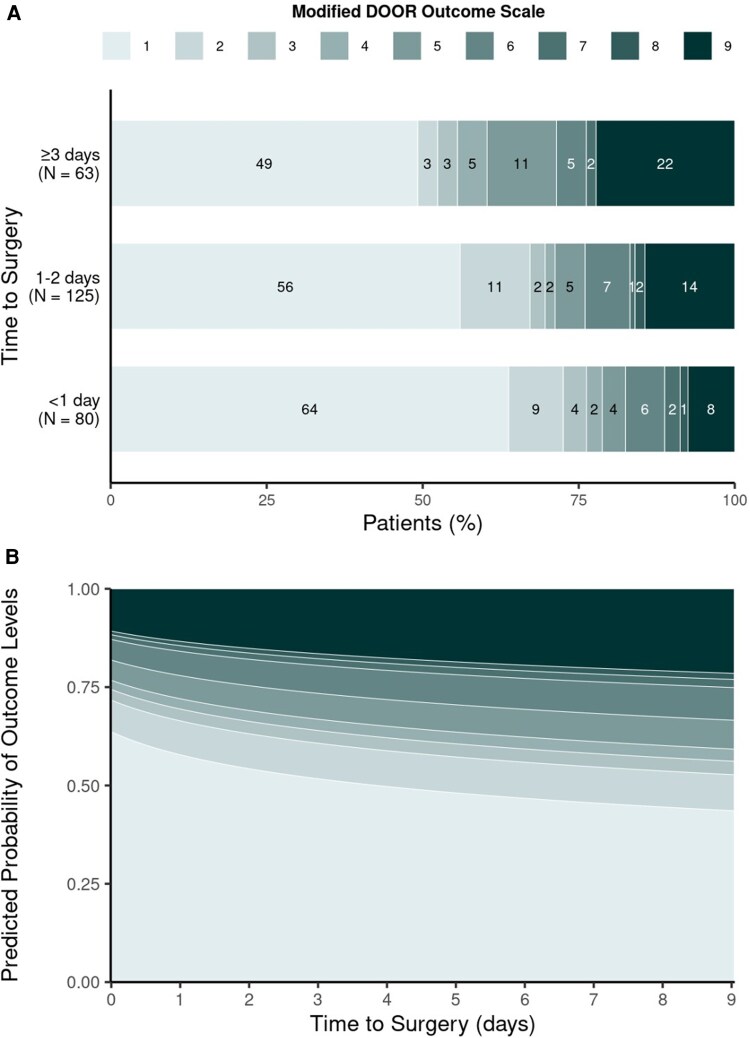
Association between time to surgery and DOOR scores at 1 year. (*A*) Distribution of modified DOOR scores by surgical timing. (*B*) Modeled probability of door scores by time to surgery.

Table 4 summarizes the risk of clinical outcomes according to surgical delay from hospital admission, both as a continuous function and as a categorical 3-level variable. Unadjusted analyses showed that longer delay, when viewed on a continuum, was significantly associated with higher (worse) DOOR scores (OR per IQR increase, 1.5; 95% CI, 1.0–2.1; *P* = .026). The overall difference in DOOR across time intervals did not reach statistical significance (*P* = .082), however, despite patients in the ≥3-day group having a 2-fold higher odds of a worse outcome compared with those in the <1-day group (OR, 2.1; 95% CI, 1.1–3.9). Figure 1 displays (A) the observed risk of DOOR ordinal outcome levels according to time intervals of surgical delay, as well as (B) the modeled risk (assuming proportional odds) as a continuous function of time within the range from 0 to 9 days.

**Table 4.** Associations Between Duration and Outcomes

	Duration Categories				Continuous	
1-Y outcome	<1 d (*N* = 80)	1–2 d (*N* = 125)	≥3 d (*N* = 63)	*P*	per IQR Δ in delay	*P*
DOOR outcome scale	…	…	…		…	…
Median (IQR)	1 (1–3)	1 (1–5)	2 (1–6)		…	…
Unadjusted OR (95% CI)	Reference	1.4 (0.8–2.5)	2.1 (1.1–3.9)	.082	1.5 (1.0–2.1)	.026
Adjusted OR (95% CI)	Reference	1.2 (0.7–2.1)	1.6 (0.8–3.1)	.376	1.3 (0.9–1.9)	.109
Mortality	…	…	…		…	…
Rate, No. (%)^a^	6 (7%)	18 (14%)	14 (22%)		…	…
Unadjusted HR (95% CI)	Reference	2.0 (0.8–5.1)	3.3 (1.3–8.5)	.038	1.7 (1.1–2.6)	.019
Adjusted HR (95% CI)	Reference	1.3 (0.5–3.3)	1.9 (0.7–5.0)	.385	1.4 (0.9–2.3)	.151
Treatment failure	…	…	…		…	…
Rate, No. (%)^a^	22 (27%)	41 (33%)	29 (46%)		…	…
Unadjusted HR (95% CI)	Reference	1.3 (0.7–2.1)	2.0 (1.2–3.5)	.038	1.5 (1.1–2.0)	.007
Adjusted HR (95% CI)	Reference	1.2 (0.7–2.0)	1.8 (1.0–3.1)	.097	1.5 (1.1–2.0)	.016
Resection/Arthroplasty	…	…	…		…	…
Rate, No. (%)^a^	8 (10%)	15 (13%)	10 (17%)		…	…
Unadjusted HR (95% CI)	Reference	1.3 (0.5–3.0)	1.9 (0.8–4.8)	.390	1.4 (0.9–2.4)	.151
Adjusted HR (95% CI)	Reference	1.3 (0.6–3.2)	2.0 (0.8–5.1)	.370	1.5 (0.9–2.4)	.136
Qualified recovery	…	…	…		…	…
Rate, No. (%)	15/74 (20%)	27/107 (25%)	9/49 (18%)		…	…
Unadjusted OR (95% CI)	Reference	1.3 (0.6–2.7)	0.9 (0.4–2.2)	.562	0.9 (0.6–1.5)	.798
Adjusted OR (95% CI)	Reference	1.3 (0.6–2.7)	0.8 (0.3–2.1)	.539	0.9 (0.6–1.5)	.723

Abbreviations: CI, confidence interval; HR, hazard ratio; IQR, interquartile range; OR, odds ratio.

^a^Rate refers to the 1-year event rate for the outcome as calculated by the Kaplan-Meier method.

Crude and adjusted effects (eg, odds ratio) were derived from unadjusted and covariate (age and CCI) adjusted semiparametric regression models; specifically, ordinal logistic (proportional odds), binary logistic, or Cox (proportional hazards) models were fitted for outcomes, and two models were fit for each to allow for analysis of duration as both a categorical and continuous predictor variable. For continuous duration, odds/hazard ratios are calculated comparing third with first quartiles (ie, 2 vs 0 days). *P* values for effects were from model-based likelihood ratio tests.

**Figure 2.**
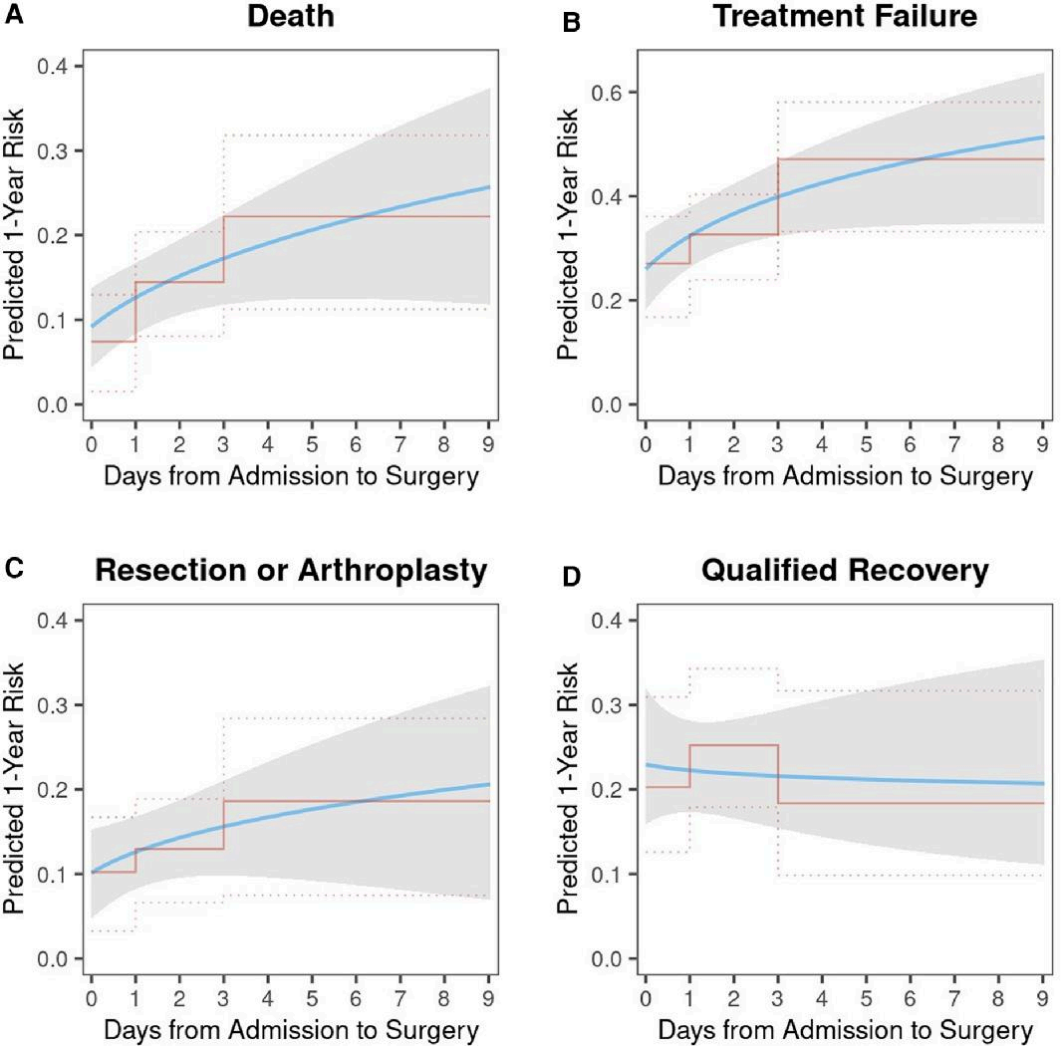
Partial effects of duration from admission to surgery on unadjusted risk of 1-year outcomes: (*A*) Death, (*B*) Treatment failure, (*C*) Resection or arthroplasty, and (*D*) Qualified recovery. Each panel shows how the estimated risk of the corresponding outcome changes as a function of surgical delay when modeled as a continuous variable (blue line) with associated 95% confidence bands (gray shaded area) and when modeled as a 3-level categorical variable (center red line) with associated 95% confidence bands (dotted red lines).

The continuous analyses demonstrated that longer surgical delay was also associated with increased 1-year mortality (HR per IQR, 1.7; 95% CI, 1.1–2.6; *P* = .019) and treatment failure (HR per IQR, 1.5; 95% CI, 1.1–2.0; *P* = .007). When the continuous measure was replaced in these models by the categorized delay variable, similar unadjusted associations were detected for mortality and treatment failure (both *P* = .038), with significantly higher risk for the patients undergoing delayed surgery ≥3 days (but not 1–2 days) as compared with <1 day. Figure 2 visualizes the unadjusted effects of surgical delay on absolute risk of secondary outcomes, with the risk relationships resulting from continuous (smooth) and categorical (step-function) effects of delay showing relatively consistent results.

In adjusted models controlling for age and comorbidity burden (as measured by the CCI), the associations between surgical delay and both the DOOR and mortality outcomes were no longer statistically significant (*P* = .109 and *P* = .151, respectively). However, the association between surgical delay (on a continuous scale) and treatment failure remained statistically significant after adjustment (adjusted HR per IQR, 1.5; 95% CI, 1.1–2.0; *P* = .016). Surgical delay was not significantly associated with qualified recovery in either unadjusted (*P* = .798) or adjusted analyses (*P* = .723).

## DISCUSSION

In this multicenter cohort study of 268 adults with NJSA, delayed surgical intervention was found to be associated with an increased risk of treatment failure. Notably, the 1-year failure rate in patients who underwent surgery ≥3 days after admission was 46%, which is nearly two-fold higher than the rate (27%) in those treated within 24 h. When considering the risk from longer delay on a continuous scale, this association remained significant even after adjustment for age and CCI, reinforcing the importance of early surgical source control. These findings provide real-world evidence supporting current recommendations favoring timely surgical intervention.

Our findings align with and expand upon prior literature demonstrating the clinical consequences of surgical delay. Vispo Seara et al. evaluated 88 patients treated arthroscopically and reported that earlier surgical intervention was associated with improved long-term functional outcomes, with 61% achieving excellent or good results and 19% classified as poor [12]. Similarly, Balabaud et al. identified surgical delay as a key predictor of treatment failure in a retrospective series of 40 patients with NJSA, defined as the persistence of local or systemic signs of infection and failure to normalize inflammatory markers [11]. These findings support expert consensus and clinical guidelines recommending prompt surgical debridement to minimize joint damage and optimize outcomes [4, 8, 9]. In contrast, other studies did not observe such associations. Kodumuri et al. assessed surgical delays in hours among 82 patients and found no significant differences in short-term outcomes, including mortality, intensive care unit admission, or need for repeat washouts [25]. Likewise, Lauper et al. analyzed 204 patients with culture-proven NJSA and found no association between surgical delay and long-term sequelae, including functional limitations or need for further surgery [26]. These conflicting findings likely reflect differences in study design, timing thresholds, definitions of treatment failure, and sample size limitations. The absence of large-scale prospective trials and standardized outcome metrics has further contributed to ongoing uncertainty in the field.

The rationale for early debridement in NJSA is supported by its potential to decompress the joint, facilitate the removal of bacterial toxins, proteases, and purulent fluid, and thereby reduce intra-articular bacterial load while limiting cartilage damage [7, 8, 10]. The biological urgency of timely intervention is illustrated in an experimental rabbit model, where *S. aureus* infection led to progressive cartilage degradation, glycosaminoglycan depletion, and ultimately severe joint destruction over time [27]. These findings underscore the rapid and irreversible nature of joint damage in the absence of intervention, highlighting the critical importance of timely source control. In select cases, early debridement may not be possible due to medical illness that precludes immediate surgery or surgeon availability. In those cases, there may be a greater role for prompt and repeated joint drainage and antimicrobial therapy as a “bridge” to definitive surgical intervention.

To evaluate the impact of timing within a multidimensional framework, we applied the DOOR ordinal outcome scale—originally developed to expand the information content of outcomes beyond binary endpoints in infectious diseases. Within the field of musculoskeletal infections, DOOR has recently been adapted for PJI [23, 28, 29]. Johns et al. developed a PJI-specific DOOR using a Delphi process involving orthopedic and infectious disease experts, identifying patient-reported joint function, infection cure, mortality, and quality of life as key domains [23]. Manning et al. used a 7-point DOOR scale in a randomized trial comparing short- versus standard-course intravenous antibiotics for PJI managed with debridement and implant retention (DAIR), demonstrating a 59.7% probability of greater desirability with short-course therapy [28]. Most recently, Johns et al. used the DOOR-PJI framework in a large prospective study of 533 patients and found that DAIR was favored in early infections, while two-stage revision was superior in late presentations [29].

Given the conceptual overlap between PJI and NJSA, such as the risk of infection recurrence and the importance of long-term joint function, the application of the DOOR framework to NJSA is a clinically relevant extension. Through expert consensus among orthopedic infectious diseases specialists and surgeons, we defined NJSA-specific outcome rankings that reflect both infection-related and functional endpoints. To our knowledge, this is the first study to apply a DOOR-based framework to NJSA, allowing for a more nuanced assessment of treatment outcomes.

The strengths of our study include its multicenter design across multiple Mayo Clinic sites, extended 1-year follow-up, and use of a structured DOOR framework. Unlike earlier studies that focused on short-term outcomes, our approach captured a broad spectrum of clinically meaningful endpoints, including infection recurrence, significant surgical events, functional recovery, and mortality. This comprehensive outcome assessment offers a more granular and patient-centered evaluation of clinical consequences beyond conventional binary measures of success or failure.

Several limitations must be acknowledged. First, this analysis included only patients who underwent surgical intervention and did not evaluate those managed medically. Surgical decision-making and access to operative care may vary across institutions, limiting generalizability. Our multicenter U.S. cohort was also characterized by a high prevalence of diabetes, chronic kidney disease, obesity, and pre-existing arthropathy, which may differ from non-U.S. populations. In addition to systemic risk factors, local factors such as pre-existing arthropathy, particularly osteoarthritis, may also predispose patients to NJSA, representing a locus minoris resistentiae.

Another limitation relates to the measurement of surgical timing. We focused on the interval from presentation to surgery, as symptom duration before admission was inconsistently documented in the medical record. Reasons for surgical delay were also variably recorded, which limited their inclusion in adjusted analyses. Our retrospective analysis was not designed to evaluate differences in outcomes within the first 24 h after presentation. While prompt surgical intervention remains biologically rational, the optimal timing of debridement should also be informed by the clinical context, including the expertise of the operating surgeon. Thus, intervention within 24 h should be regarded as urgent, yet balanced with the availability of appropriate surgical expertise to achieve the best outcomes. Although we adjusted for age and comorbidity, additional factors such as bacteremia, sepsis, and infection site were not incorporated, and a small number of small-joint infections were included, which may differ in prognosis from large joints.

Interestingly, surgical delay was not significantly associated with functional recovery in our cohort. However, this finding should be interpreted with caution. Functional recovery in our study was assessed based on clinical documentation rather than more comprehensive, objective joint function scores, such as the Oxford Hip and Knee Scores [30, 31], or validated patient-reported outcomes [32], which were unavailable due to the retrospective nature of the study. Furthermore, our analysis was also limited by the lack of adjustment for variables that could influence recovery, including baseline joint status, intraoperative findings, quality of debridement, and access to rehabilitation. Future prospective studies should incorporate these variables and validated functional assessments to better characterize long-term outcomes.

In addition, while the DOOR framework was originally developed for use in clinical trials, its application in retrospective studies remains limited. In this study, the DOOR ranking system was adapted through internal consensus among orthopedic infectious diseases specialists and an orthopedic surgeon to reflect the clinical context of NJSA. Its validity and interpretability in retrospective settings has yet to be fully established. Broader consensus and further evaluation in larger, prospective studies are needed to confirm its utility.

Lastly, although mortality was categorized as the worst outcome within the DOOR framework, our analysis did not distinguish between infection-related and non-infection-related deaths. This approach aligns with conventional DOOR framework models but may overrepresent the direct impact of infection to mortality. Conversely, excluding non-infectious deaths introduces the risk of selection bias by censoring the most severe outcomes.

Despite these limitations, this study provides critical insights into the clinical impact of surgical timing in NJSA and introduces a novel application of the DOOR framework to NJSA. Our findings reinforce the value of early surgical source control and emphasize the value of multidimensional approach in evaluating patient outcomes. Further prospective studies are warranted to validate these findings and guide clinical decision-making.

## Supplementary Material

ofaf662_Supplementary_Data

## Supplementary Data

Supplementary materials are available at *Open Forum Infectious Diseases* online. Consisting of data provided by the authors to benefit the reader, the posted materials are not copyedited and are the sole responsibility of the authors, so questions or comments should be addressed to the corresponding author.
