# Supplementary material for: Consequences of Delaying Surgical Intervention in Patients With Native Joint Septic Arthritis
Source: Open Forum Infect Dis. 2025 Oct 24;12(11):ofaf662. doi: 10.1093/ofid/ofaf662 (PMC12605720; doi:10.1093/ofid/ofaf662)
Supplement: ofaf662_Supplementary_Data [file ofaf662_supplementary_data.docx]

**Supplementary Figure 1. Assessment of the proportional odds assumption for the surgical delay predictor**

The proportional odds (PO) assumption dictates that the odds ratio for the time to surgery effect does not depend on the outcome level of the ordinal scale. When this assumption holds, the odds ratio estimated from the ordinal logistic model will be an appropriate summary measure of effect since it represents an average of the odds ratio results obtained from separate binary logistic models. Each panel in the figure below considered a different functional form of delay: (A) nonlinear effect of log time (using a 4-knot regression spline), (B) linear effect of log time, and (C) categorical effect. Odds ratios were calculated by comparing the third with first quartiles (i.e., 2 vs 0 days) when delay entered the analysis as a continuous variable, and by comparing each interval with a reference interval (<1 day) for categorized delay. The patterns depicted below show that the PO assumption is better satisfied when delay is modeled as linear in log time rather than as nonlinear (or as categorical). Because there was little to no evidence of nonlinear effects of log time on outcomes, and because the model assumptions of proportional odds/hazards tended to be better satisfied when re-fitted as linear, we modeled the continuous-scale delay variable without the use of a spline for the final outcome analyses.


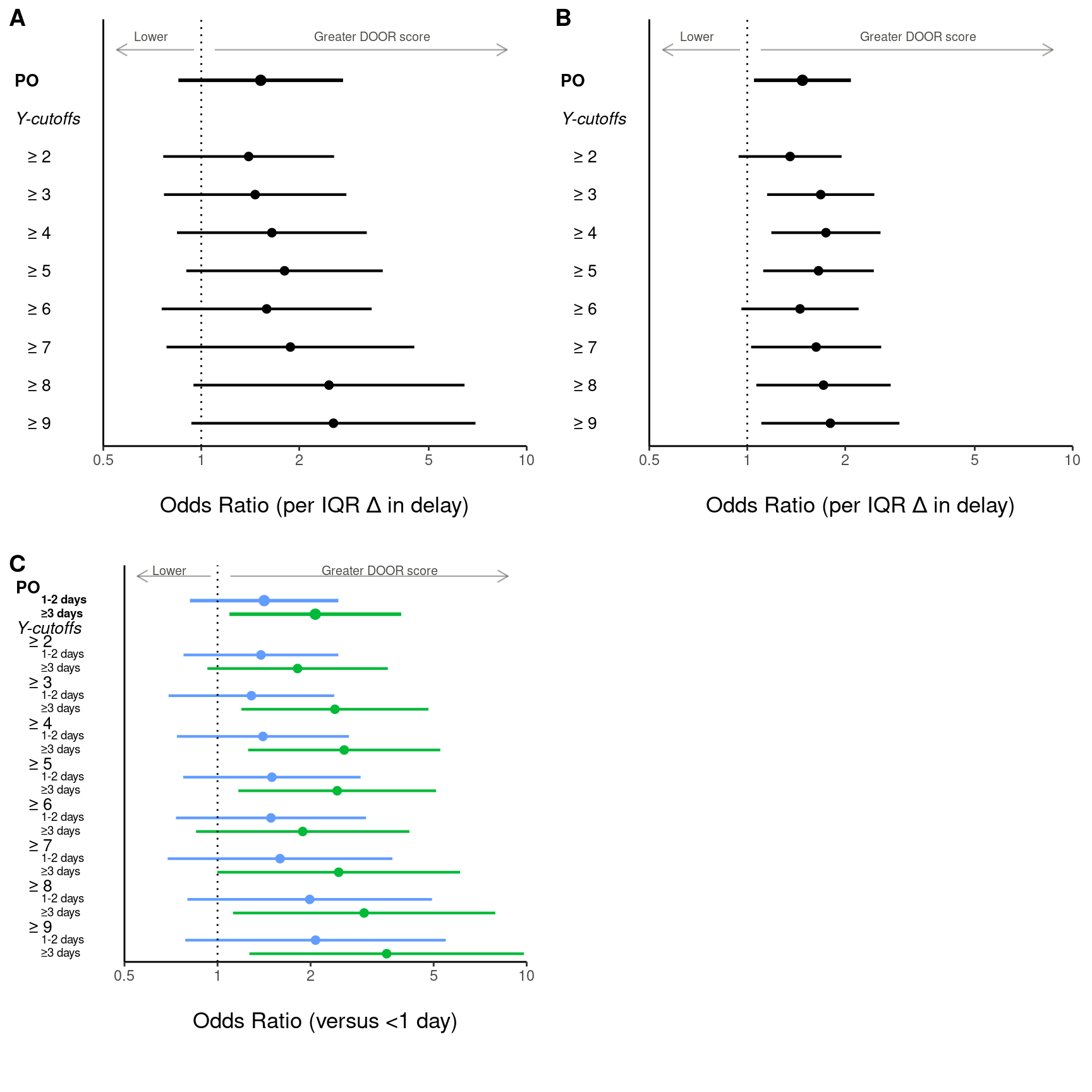


**Supplementary Table 1. Reasons for Delayed Surgical Intervention (≥3 days after presentation, n = 63)**

| **Reason for Delay** | **n** | **%** |
| --- | --- | --- |
| Diagnostic uncertainty (further evaluation required) | 20 | 31.7 |
| Hemodynamic instability | 12 | 19.0 |
| Prioritized management of concurrent infection (e.g., endocarditis, discitis) | 8 | 12.7 |
| Orthopedic surgeons’ decision / surgical scheduling issues | 6 | 9.5 |
| Initial medical management followed by failure | 3 | 4.8 |
| Anticoagulation requiring reversal | 2 | 3.2 |
| Unknown / not documented | 12 | 19.0 |
